# Resveratrol Butyrate Esters Inhibit BPA-Induced Liver Damage in Male Offspring Rats by Modulating Antioxidant Capacity and Gut Microbiota

**DOI:** 10.3390/ijms22105273

**Published:** 2021-05-17

**Authors:** Jin-Xian Liao, Yu-Wei Chen, Ming-Kuei Shih, You-Lin Tain, Yao-Tsung Yeh, Min-Hsi Chiu, Sam K. C. Chang, Chih-Yao Hou

**Affiliations:** 1 Department of Seafood Science, National Kaohsiung University of Science and Technology, Kaohsiung 811, Taiwan; j0920181@gmail.com; 2 Department of Medicine, Chang Gung University, Linkow 333, Taiwan; naosa720928@gmail.com; 3 Graduate Institute of Food Culture and Innovation, National Kaohsiung University of Hospitality and Tourism, 812301 No.1, Songhe Rd., Xiaogang Dist., Kaohsiung 833, Taiwan; mkshih@mail.nkuht.edu.tw; 4 Department of Pediatrics, Kaohsiung Chang Gung Memorial Hospital and Chang Gung University College of Medicine, Kaohsiung 833, Taiwan; tainyl@hotmail.com; 5 Institute for Translational Research in Biomedicine, Kaohsiung Chang Gung Memorial Hospital and Chang Gung University College of Medicine, Kaohsiung 833, Taiwan; 6 Aging and Disease Prevention Research Center, Fooyin University, Kaohsiung 83102, Taiwan; glycosamine@yahoo.com.tw (Y.-T.Y.); c12010521@yahoo.com.tw (M.-H.C.); 7 Biomed Analysis Center, Fooyin University Hospital, Pingtung 92849, Taiwan; 8 Experimental Seafood Processing Laboratory, Costal Research and Extension Center, Mississippi State University, Pascagoula, MS 39567, USA; schang@fsnhp.msstate.edu; 9 Department of Food Science, Nutrition and Health Promotion, Mississippi State University, Starkville, MS 39762, USA

**Keywords:** resveratrol butyrate esters (RBE), maternal/fetal animal model, bisphenol A (BPA), gut microbiota, short-chain fatty acids (SCFAs), offspring

## Abstract

Resveratrol can affect the physiology or biochemistry of offspring in the maternal–fetal animal model. However, it exhibits low bioavailability in humans and animals. Fifteen-week SD pregnant female rats were orally administered bisphenol A (BPA) and/or resveratrol butyrate ester (RBE), and the male offspring rats (n = 4–8 per group) were evaluated. The results show that RBE treatment (BPA + R30) compared with the BPA group can reduce the damage caused by BPA (*p* < 0.05). RBE enhanced the expression of selected genes and induced extramedullary hematopoiesis and mononuclear cell infiltration. RBE increased the abundance of *S24-7* and Adlercreutzia in the intestines of the male offspring rats, as well as the concentrations of short-chain fatty acids (SCFAs) in the feces. RBE also increased the antioxidant capacity of the liver by inducing Nrf2, promoting the expression of HO-1, SOD, and CAT. It also increased the concentration of intestinal SCFAs, enhancing the barrier formed by intestinal cells, thereby preventing BPA-induced metabolic disruption in the male offspring rats, and reduced liver inflammation. This study identified a potential mechanism underlying the protective effects of RBE against the liver damage caused by BPA exposure during the peri-pregnancy period, and the influence of the gut microbiota on the gut–liver axis in the offspring.

## 1. Introduction

Endocrine disrupting chemicals (EDCs), also referred to as environmental hormones, are exogenous compounds that interfere with the endocrine system and demonstrate adverse effects in organisms [1]. Among the wide variety of EDCs, bisphenol A (BPA) is the most prevalent in the environment [2]. Rodríguez-Carrillo et al. reported that BPA may predominantly affect the behavior of children [3]. Studies conducted in Europe and the United States have reported the presence of detectable levels of BPA in more than 90% of urine samples in the general population [4,5,6]. Long-term exposure to BPA may increase the risks of obesity, diabetes mellitus, and breast cancer [7,8]. Previous studies demonstrated the developmental origins of the health and disease (DOHaD) hypothesis [9,10], which highlighted the link between the periconceptual, fetal, and early infant phases of life and the subsequent development of adult obesity and related metabolic disorders [11,12]. Associated research has also indicated that exposure to a functional compound may reprogram the adult offspring’s metabolism to cause metabolic syndrome, induced maternally by different medical effects (such as EDCs).

Resveratrol (RSV) has been demonstrated to be involved in a variety of bioactivities and functions, such as the reduction of oxidative damage and inflammation, neuroprotection, chemoprevention, anti-obesity effects, and anti-aging effects [13,14,15,16,17,18]. Previous research has also shown that perinatal BPA exposure increases the risk of cardiometabolic diseases [19] and liver injury [20] in mouse offspring, and affects hypothalamic neuronal growth and appetite in rat offspring [21]. Supplementation with RSV during the perinatal period influences the physiology and health of the offspring [22,23,24]. Kubo et al. (2003) reported that RSV reduced the adverse effects of perinatal BPA exposure on the locus coeruleus and reproductive systems of rat offspring [25]. In our previous study, we found that the kidneys of the male offspring of rats perinatally exposed to BPA and a high-fat diet could be protected by RSV treatment, which exerted its effects by restoring the bioavailability of NO, reducing oxidative stress, and antagonizing the AHR signaling pathway [26].

RSV therapy can increase the α-diversity, decrease the Firmicutes-to-Bacteroidetes ratio, and increase the abundance of the gut microbiota [27,28]. Although RSV demonstrates various physiological activities and functions, its bioavailability is extremely low [29,30,31,32,33,34]. For instance, only a trace amount of RSV (<5 ng/mL) can be detected in the plasma following the oral administration of 25 mg of RSV [35]. Therefore, attempts to increase the bioavailability of RSV, such as research on RSV derivatives, are crucial. Oh et al. reported that the esterification of RSV led to higher lipophilicity and better antioxidant effects in a lipophilic system [36]. Another study by Hu et al. found that RSV esters were hydrolyzed into free RSV and fatty acids by pancreatic lipase in an in vitro gastrointestinal digestion system [37]. Therefore, in addition to serving as potent antioxidant agents in fat-soluble foods, RSV esters can also function as carriers of specific fatty acids to prevent their oxidation during storage and enable the gradual release of the fatty acids in the gastrointestinal tract. In a previous study, we successfully enhanced the production and activity of the products of a Steglich esterification reaction [38] with *N*-ethyl-*N*’-(3-dimethylaminopropyl) carbodiimide (EDC) and 4-dimethyl aminopyridine (DMAP). When the reaction was performed for the esterification of RSV and butyric acid to form resveratrol butyrate esters (RBEs), a 30% increase in the production of RBE was observed [39]. In addition, in the previous study, we reported novel resveratrol butyrate esters (RBE) and indicated that the esterification of RSV with butyrate produced RSV, RBE monoester, and RBE diester, which also showed better hydrogen peroxide-scavenging activity than RSV [40]. It was also found that RBE can effectively inhibit fatty acid-induced lipid accumulation in HepG2 cells, with effects similar to those of RSV achieved at a lower dose [39]. Therefore, RBE can potentially serve as a functional food ingredient and supplement for health promotion.

Short-chain fatty acids (SCFAs) are the end products of the fermentation of food performed by the intestinal microbiota, with acetic acid, propionic acid, and butyric acid comprising 95% of SCFAs in the human gut [41]. These SCFAs not only serve as a source of energy for the host, but also participate in many metabolic processes in the human body, such as energy metabolism [42], the inflammatory response [43], adipose tissue formation [44], and liver metabolism [45]. In addition, SCFAs are also beneficial in disease treatment, because they can effectively inhibit colon cancer cell proliferation and provide energy to normal colonic epithelial cells [41]. In a study on hypertension, it was found that SCFAs attenuated increases in blood pressure via the regulation of steroid metabolism [46]. Among various SCFAs, butyric acid has been considered to be the most important, with many studies indicating that butyric acid can effectively regulate the apoptotic pathway in cells, prevent colon cancer, reduce bacterial translocation and the inflammatory response, and improve the intestinal barrier function [47,48]. Butyric acid also modulates mitochondrial function, including the enhancement of oxidative phosphorylation and β-oxidation, and has been demonstrated as a neuroprotectant that alleviates mitochondrial dysfunction in patients with autism spectrum disorders [49]. Butyric acid influences the formation and differentiation of gut microbiota, with many studies indicating that timely supplementation with butyric acid can effectively regulate the Firmicutes/Bacteroidetes (F/B) ratio of gut microbiota and promote an increase in Akkermansia abundance, which is beneficial for the prevention of health problems such as obesity, atherosclerosis, and insomnia [46]. Butyric acid is usually taken in the form of butyrate salts. In recent years, researchers have reported the use of butyrate esters for butyric acid supplementation, which prevents the side effect of hypernatremia. In addition, butyrate esters demonstrate higher stability and a longer half-life in the body than butyric acid, and can be hydrolyzed into butyric acid by lipases [50]. Supplementation with butyrate esters can result in a four-fold increase in the final butyric acid content detected in the plasma of organisms [50,51,52].

To facilitate a wide range of applications and enhance bioactivity, we exposed maternal rats to BPA combined with RSV or RBE to investigate the effects of perinatal RBE supplementation on liver damage and gut microbiota of the male offspring. This study is the first animal experiment to explore, particularly during the peri-pregnancy period, the effects of oral RBE on plasma biochemical markers and liver function protection in male offspring rats (born from the female rats that were exposed to BPA), and to further analyze the relationship between the growth and decline of the gut microbiota of the male offspring.

## 2. Results

### 2.1. Changes in the Plasma Biochemical Markers in Male Offspring Rats

Perinatal BPA exposure in the maternal rats led to a significant increase in the levels of liver function indicators, blood lipids, and malondialdehyde (MDA) in the male offspring rats (*p* < 0.05). In the BPA + R30 group, all of the negative effects of BPA were attenuated (*p* < 0.05), with an increase in HDL (238.9 μg/mL) and decrease in MDA levels (173.1 pg/mL) compared with those observed in the control group (*p* < 0.05). We found that the ALT (51.5 U/L) and AST (101.7 U/L) of the BPA + R30 groups showed increases of approximately 18% and 37%, respectively, compared with those of the control group, although this was significantly lower than that in the BPA (without R30) groups (approximately 75% and 99%, respectively). This proves that RBE affects liver metabolism. Similar results were also observed for BW, TG, and MDA. Overall, the results indicate that supplementation with R30 (RBE) provided full protection against perinatal BPA exposure in the male offspring rats.

### 2.2. Gene Expression and Enzymatic Activities of Antioxidant Enzymes in the Liver Tissues of Male Offspring Rats

**Figure 1:**
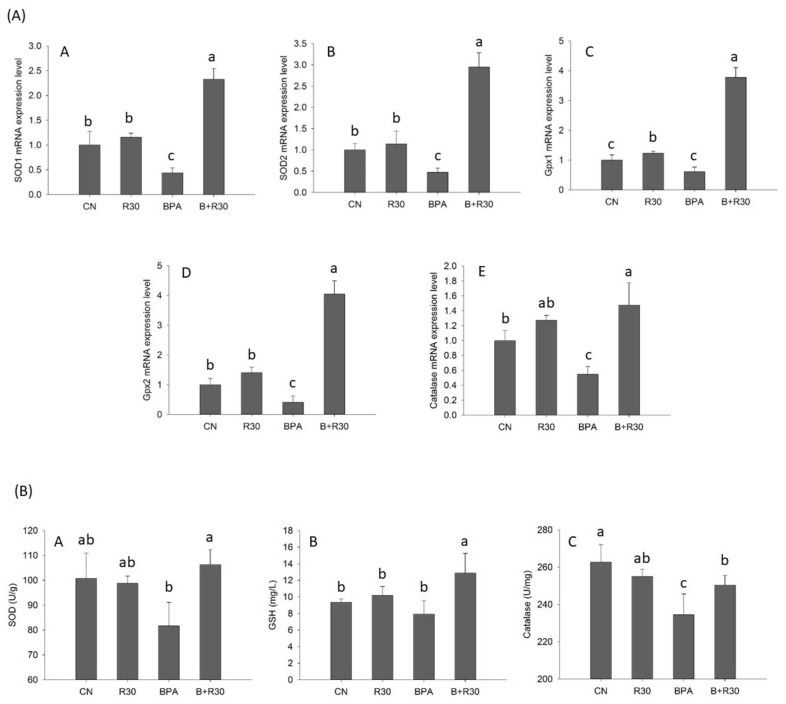
(**A**) Effects of bisphenol A (BPA) and resveratrol ester on gene expression in the livers of male offspring rats. A: SOD1 gene expression in the liver. B: SOD2 gene expression in the liver. C: Gpx1 gene expression in the liver. D: Gpx2 gene expression in the liver. E: Catalase gene expression in the liver. (**B**) Effects of BPA and resveratrol ester on antioxidant enzyme activity of male offspring rats. A: SOD activity (U/g) in male offspring rats. B: GSH (mg/L) in male offspring rats. C: Catalase activity (U/mg) in male offspring rats. *n* = 4–8 per group; ^a–c^ *p* < 0.05, significantly different from other groups. CN = control group; R30 = group administered 30 mg/kg/day of resveratrol butyrate ester (RBE); BPA = group administered bisphenol A at 50 μg/kg/day; BPA + R30 = group administered 30 mg/kg/day of RBE and bisphenol A.

When the mRNA expression and enzymatic activities of the selected antioxidant genes were analyzed, it was found that, with the exception of the mRNA expression of Gpx1, all the gene expression levels (SOD1, SOD2, Gpx2, and catalase) were significantly reduced in the liver tissues of the male offspring of the maternal rats with perinatal BPA exposure (*p* < 0.01). RBE supplementation restored or increased the expression of all the selected genes (Figure 1A). The effects observed in the BPA + R30 group were significantly greater than those observed in the R30 group for all the gene expression levels except for catalase mRNA expression. From analyzing the BPA + R30/CN and R30/CN gene expression ratios for SOD1, SOD2, Gpx1, Gpx2, and catalase, it can be observed that the BPA + R30 group also demonstrated significantly greater expression of all the selected genes than the R30 group. This observation indicates that certain effects were exerted by BPA/RBE treatment in the male offspring rats, which led to differences in the induction of the expression of selected genes compared with that observed with the use of RBE alone. The measured activities of the selected enzymes (Figure 1B) showed that BPA significantly decreased the catalase activity in the offspring *p* < 0.01). However, a decrease in SOD and catalase activities and GSH content after BPA exposure was significantly recovered after RBE supplementation. Similarly to the results for mRNA expression, except for catalase activity, the effects of BPA/RBE on SOD activity and GSH content were stronger than those of RBE alone. Owing to the increase in ALT and AST activity in the blood (Table 1), we suggest that RBE may attenuate liver damage by increasing the activities of antioxidant enzymes in the liver.

### 2.3. Hematoxylin and Eosin (H&E) and Immunohistochemical (IHC) Staining of Liver Tissue Sections of Male Offspring Rats

**Figure 2:**
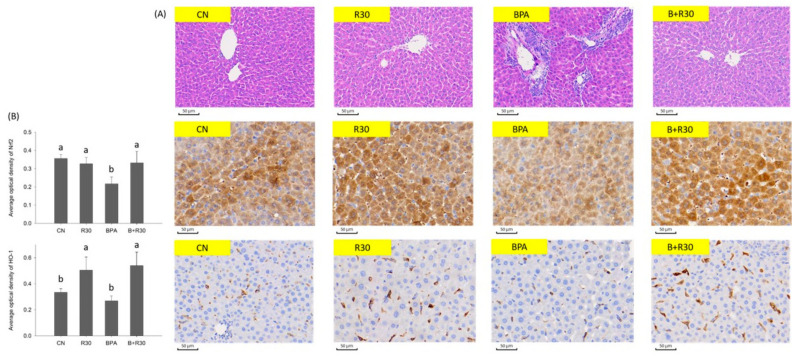
(**A**) Representative pictures of the liver sections stained with hematoxylin and eosin (H&E) examined under a microscope. (**B**) Expression of Nrf2 and HO-1 observed in liver immunohistochemistry of male offspring rats. *n* = 4–8 per group; ^a–b^ *p* < 0.05, significantly different from other groups. CN = control group; R30 = group administered 30 mg/kg/day of RBE; BPA = group administered bisphenol A at 50 μg/kg/day; BPA + R30 = group administered 30 mg/kg/day of RBE and bisphenol A.

**Figure 3:**
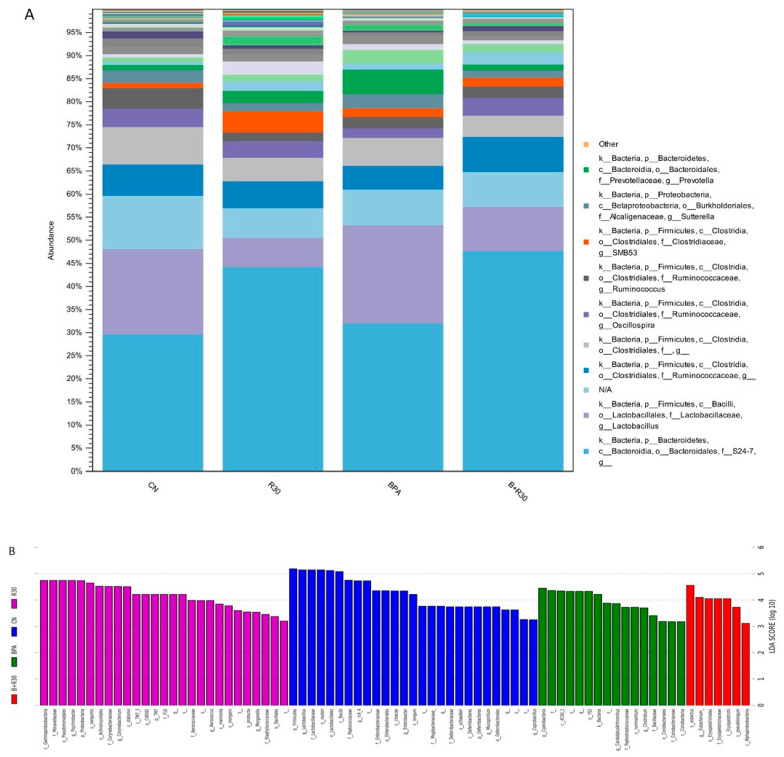

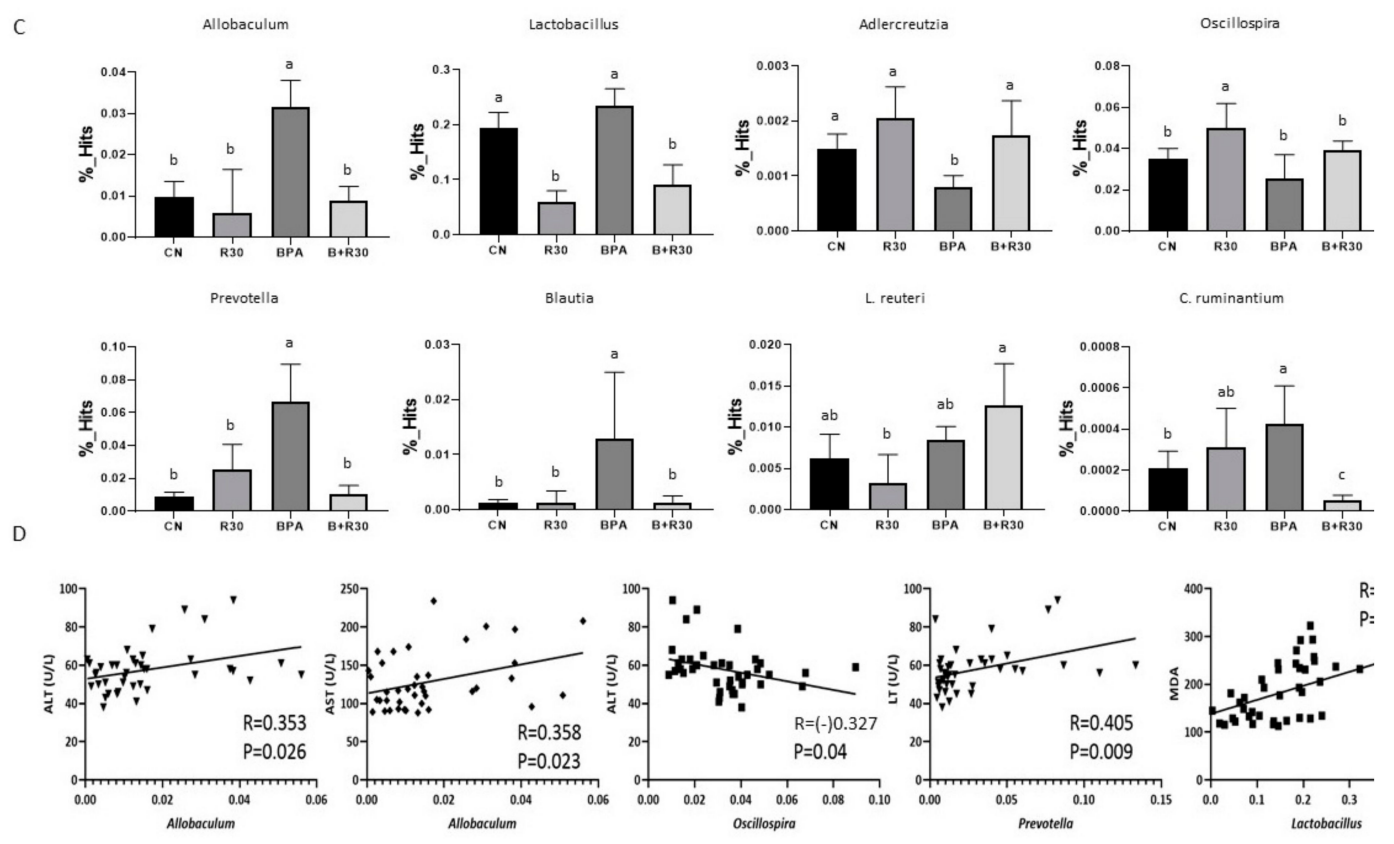
Effect of BPA and RBE on the gut microbiota of male offspring rats. (**A**) The gut bacterial composition at the phylum level. (**B**) Biomarker taxa generated from LEFSe analysis (LDA > 3). (**C**) Comparison of the growth and decline of specific strains. (**D**) The correlation analysis of bacterial species and plasma biochemical indicators. *n* = 4–8 per group; ^a–b^ *p* < 0.05, significantly different from other groups. CN = control group; R30 = group administered 30 mg/kg/day of RBE; BPA = group administered bisphenol A at 50 μg/kg/day; BPA + R30 = group administered 30 mg/kg/day of RBE and bisphenol A.

Angiogenesis and the staining of Nrf2/HO-1 were analyzed in the liver tissue sections of the male offspring rats. H&E staining revealed that BPA induced extramedullary hematopoiesis around the hepatic portal vein and mononuclear cell infiltration in the male offspring rats. This indicates that BPA exposure in maternal rats during pregnancy may result in the development of a hepatic inflammatory response in the male offspring. However, RBE supplementation attenuated this phenomenon (Figure 2A). This is consistent with the previous experimental result and demonstrates the capability of RBE in alleviating inflammatory responses in the liver, reducing plasma AST and ALT activities, and effectively increasing the activities of antioxidant enzymes in the body, thereby preventing inflammation. When IHC staining was performed for the observation of the distribution of Nrf2 and HO-1 in the liver, it was found that Nrf2 was significantly decreased in the liver sections of the BPA group (Figure 2B). This indicated that BPA decreased Nrf2 expression and, consequently, increased oxidative stress in the liver regardless of whether it was directly administered to the offspring rats or indirectly administered through the maternal rats. Our results also show that Nrf2 expression was restored to normal levels in both the RBE and BPA/RBE groups. BPA significantly inhibited HO-1 expression; however, the inhibitory effects were attenuated by RBE supplementation (Figure 3C). Once again, the IHC stains showed that the enhancing effects of BPA/RBE on HO-1 expression were significantly higher than those of RBE alone (*p* < 0.05). The results of the IHC staining suggest that Nrf2 promoted HO-1 transcription and increased protein expression in the liver after entry into the cell nucleus.

Because RSV enhances the antioxidant capacity of the liver by inducing Nrf2/HO-1 expression, it can be reasonably deduced that certain effects of RSV were preserved in RBE after esterification, which offset the damaging effects of BPA. It is also possible that the digestion and metabolic products of RBE directly or indirectly altered the physiological conditions of the maternal rats, thereby influencing gene regulation in the liver tissues of the offspring. The results described above reveal that RBE promoted HO-1 transcription and SOD and CAT expression by increasing Nrf2 expression. Consequently, the antioxidant capacity of the liver was enhanced, thereby protecting against the oxidative damage induced by BPA.

### 2.4. Changes in Intestinal Flora Type and Abundance, and SCFA Concentration in the Guts of Male Offspring Rats

An analysis of fecal samples revealed a difference in the abundance of the intestinal flora of the male offspring rats treated with BPA/RBE. Figure 3A shows the relative abundance of intestinal bacteria phyla among the CN, R30, BPA, and BPA + R30 groups according to operational taxonomic unit (OTU) analysis. The observations of the microbiota succession described above indicate that an increase in the abundance of *S24-7* in both the BPA + R30 and R30 groups was accompanied by a decrease in the abundance of Prevotella (Figure 3A). The LEfSe analysis showed differences in the abundance of various bacterial species among the CN, R30, BPA, and BPA + R30 groups (Figure 3B). In addition, the analysis of the fecal samples of the male offspring revealed that the abundance of Allobaculum, Lactobacillaceae, Prevotella, and Blautia increased after BPA exposure, with an increase in *L. reuteri* and *C. ruminantium* being particularly prominent, whereas the abundance of Adlercreutzia and Oscillospira decreased (Figure 3C). With RBE supplementation (BPA + R30 group), the abundance of *S24-7* and Adlercreutzia increased significantly, whereas the abundance of Allobaculum, Blautia, Lactobacillaceae, and Prevotella decreased significantly; a decrease in *C. ruminantium* was also observed. The correlation analysis (Figure 3D) showed that Prevotella and Allobaculum were positively correlated with the plasma ALT concentration, while Oscillospira was negatively correlated with the ALT concentration. Allobaculum and *C. ruminantium* were positively correlated with the AST concentration, and Lactobacillaceae was positively correlated with the MDA concentration. The oral administration of BPA in maternal rats disrupted the composition of their gut microbiota. The results of this study further indicate that the perinatal ingestion of BPA by maternal rats also disrupts the gut microbiota in the offspring. This may be attributed to the transfer of BPA from maternal rats to offspring through umbilical cord blood during pregnancy or through breast milk during breastfeeding, which enables BPA to exert its effects on the gut microbiota of the next generation. Additionally, the BPA-induced liver damage observed in the offspring may also influence the gut microbiota distribution via the gut–liver axis.

Given the significant influence exerted by SCFAs on gut microbiota, we also analyzed the SCFA content of the fecal samples of the male offspring. Our results indicate that the concentrations of acetic acid and propionic acid in the guts of the BPA group’s male offspring increased; however, there was no significant difference in butyric acid content compared with that in the control group (Table 2). By contrast, the acetic acid, propionic acid, and butyric acid concentrations in the gut were effectively increased in the BPA + R30 group (Table 2). In addition to providing energy to intestinal epithelial cells, these metabolites can also enter the liver through the portal vein circulation, thereby providing energy and inhibiting inflammation in the liver.

## 3. Discussion

### 3.1. Effects of RBE on BPA-Induced Physiological and Biochemical Changes in Offspring Rats

BPA exposure led to significant increases in the body weight and fat mass in the offspring rats. This was mainly attributed to the fact that BPA regulates the expression of orexigenic and anorexigenic neurons by stimulating the differentiation of hypothalamic neurons, thereby increasing the appetite and energy intake in the offspring rats [21]. In addition, BPA exposure also led to increased oxidative responses in the livers of the offspring, which resulted in the impairment of the liver function [2]. Therefore, the levels of the liver function indicators ALT and AST were significantly higher in the BPA group than in the CN group. This observation is in agreement with the results of a study by Meng et al. [20], who investigated the effects of perinatal exposure to BPA, BPF, and BPAF on liver function impairment in male mouse offspring. In this study, we were the first to study the effect of RBE on BPA-induced liver damage in male offspring rats; thus, more research is needed to confirm the relevant mechanisms. In addition, because few studies have investigated the effects of combined treatment with BPA and RBE, it is difficult to deduce if their metabolic reactions offset each other’s after simultaneous ingestion. The ALT and AST concentrations were significantly decreased in the BPA + R30 group compared with those in the BPA group. The results found for ALT and AST in the R30 groups (which were approximately 18% and 37% higher, respectively, than in the control group) may suggest that RBE affects liver metabolism. However, our previous study demonstrated that RBE does not cause cytotoxicity to HepG2, according to an MTT assay [39]. In addition, RBE can inhibit hydroxyl radical-induced DNA scission [40], which indirectly demonstrates the safety of RBE. For the sake of caution, the ADME of resveratrol and RBE (including resveratrol, resveratrol monoester 1, resveratrol monoester 2, and resveratrol diester) were evaluated using the SwissADME software, which is a free web tool for evaluating pharmacokinetics, drug-likeness, and medicinal chemistry friendliness, taking six physicochemical properties into account: lipophilicity, size, polarity, solubility, flexibility, and saturation [53]. The ADME results for RSV and RBE are shown in Appendix A Table A1 and Appendix A Figure A2, and indicate that RBE displays similar safety information to that associated with RSV. In addition, no difference is observable between the RBE and CN groups in the liver tissue section results (Figure 2A). Therefore, we consider that the results for ALT and AST in the R30 group may have a different metabolic explanation. Although ALT and AST were significantly lower in the R30 groups than the BPA groups (by approximately 75% and 99%, respectively), this result still presents an effect on liver metabolism of RBE; thus, the related reasons are worthy of further research. Previous research has indicated that maternal exposure to RSV during pregnancy increased the expression of antioxidant enzymes, enhanced antioxidant capacity, and reduced the synthesis of oxidation products in the offspring [54]. Franco et al. [55] also reported that maternal exposure to RSV during pregnancy effectively increased the activities of antioxidant enzymes in the offspring’s liver and reduced the incidence of non-alcoholic fatty liver disease in the offspring.

After BPA exposure, the plasma TG and TC concentrations in the BPA group increased significantly. Previous research has shown that BPA may induce metabolic abnormalities and increase plasma TG and TC concentrations in offspring [20]. In a study conducted with mouse 3T3-L1 cells, it was found that BPA increased the activity of lipoprotein lipase (LPL), thereby increasing TG synthesis in adipocytes [56]. Our results also showed that BPA caused abnormalities in the plasma HDL and LDL levels. The BPA group demonstrated a significant decrease in HDL and significant increase in LDL. HDL and LDL play important roles in the metabolism of cholesterol and TG in the human body. HDL is responsible for the clearance of cholesterol and TG from the blood, and LDL is a transporter of cholesterol and TG [57]. Therefore, abnormalities in the HDL and LDL concentrations in the blood may result in increased TG and TC levels in the body. In the BPA + R30 group, it was observed that the plasma HDL and LDL levels were gradually restored to normal and the TG and TC concentrations also decreased. This may be attributed to the effects of RSV in stimulating energy metabolism and attenuating the hyperlipidemic symptoms induced by type 2 diabetes mellitus [58], which may have been retained in RBE after esterification, reducing the risk of cardiovascular disease.

In the BPA group, the MDA concentration significantly increased to 308.4 pg/mg. MDA is the end product of lipid peroxidation and is widely used as an indicator of oxidative stress in organisms. The excessive accumulation of reactive oxygen species leads to the oxidation of organic molecules, such as proteins, lipids, and DNA, in the body, which can result in vascular aging and atherosclerosis. Our results indicate that the MDA concentration decreased significantly in both the BPA + R30 and R30 groups, implying that the ingestion of RBE effectively reduces oxidative stress. This may have been achieved by the enhancement of the liver function and antioxidant activity of the offspring rats, thereby resulting in a decrease in the plasma MDA concentration.

### 3.2. RBE Attenuated BPA-Induced Damage by Enhancing the Expression of Selected Genes and the Activities of Antioxidant Enzymes and Proteins

Antioxidant enzymes are essential components of intracellular antioxidant mechanisms. When cells generate reactive oxygen species (ROS) through energy metabolism or immune responses, the protein expression of antioxidant enzymes is endogenously triggered for ROS clearance. External stimuli or drug administration may also increase the gene expression of antioxidant enzymes within cells [59]. In the present study, we found that BPA exposure caused a significant decrease in the gene expression of SOD1, SOD2, Gpx2, and catalase. Anet et al. [60] studied the effects of BPA exposure on oxidative damage and genotoxicity in the common fruit fly (*Drosophila melanogaster*) and found that BPA not only resulted in increased ROS and lipid peroxidation, but also reduced the protein expression of antioxidant enzymes. However, the BPA + R30 treatments effectively attenuated the BPA-induced decrease in the gene expression of antioxidant enzymes. This may be related to the ability of RBE to stimulate the entry of Nrf2 into the cell nucleus, where it binds to the antioxidant response element (ARE) and promotes the gene expression of antioxidant enzymes [61]. The activities of the selected enzymes and antioxidant protein concentrations measured in this study indicated that the differences in the decrease in SOD and GSH between the CN and R30 groups were statistically insignificant, but significant increases were observed in the BPA + R30 group compared with the levels in the BPA group. This result indicates that the simultaneous ingestion of RBE and BPA was still effective in triggering the activity of antioxidant enzymes in the male offspring rats, which is consistent with the ALT, AST, and MDA results shown in Table 1. Therefore, it is evident that RBE attenuated liver damage and peroxidation by increasing the activities of antioxidant enzymes in the liver.

Extramedullary hematopoiesis is typically observed during infancy or under conditions such as hypoxia and anemia. However, because damage to human organs caused by inflammatory responses, cancer, or organ transplantation results in the migration of hematopoietic stem cells to tissues and organs, extramedullary hematopoiesis is also regarded as an indicator of pathological changes in organs [62]. In the present study, both extramedullary hematopoiesis and mononuclear cell infiltration were observed in the BPA group, and were caused by cell migration induced by the inflammatory response. Therefore, perinatal exposure to BPA in maternal rats may elicit an inflammatory response in male offspring rats. An increase in the RBE dosage led to a decrease in symptoms, which is consistent with the experimental results described above and demonstrates that RBE indeed alleviated the inflammatory response in the liver and prevented the occurrence of inflammation by reducing the plasma AST and ALT concentrations and enhancing the activities of antioxidant enzymes.

Nrf2 is a key protein in the regulation of oxidative stress in cells. Under normal circumstances, Nrf2 binds to Keap1 and is retained in the cytoplasm. When oxidative stress increases, the Nrf2–Keap1 complex is disrupted and free Nrf2 is translocated into the cell nucleus, where it activates the ARE-mediated transcription of genes for multiple antioxidant enzymes, such as SOD, CAT, Gpx, and HO-1 [63]. Nrf2 has been indicated to be a cytoprotective factor regulating the expression of genes’ coding for anti-inflammatory, antioxidant, and detoxifying proteins and, to some extent, it is mimicked by Nrf2-dependent genes and their proteins, including heme oxygenase-1 (HO-1). In addition, HO-1 and its products exert beneficial effects via protection against oxidative injury, the regulation of apoptosis, and the modulation of inflammation, in addition to contributing to angiogenesis [64]. In the present study, we performed IHC staining to observe the distribution of Nrf2 and HO-1 in the liver tissues of the male offspring rats. The liver sections of the BPA group exhibited a significant decrease in Nrf2, which is in agreement with the results of a study conducted by Müller et al. that examined the hepatic oxidative damage caused by BPA in male mice [65]. This indicates that both direct BPA exposure by ingestion and indirect exposure through maternal ingestion led to a decrease in Nrf2 expression in the livers of the male offspring rats, which increased hepatic oxidative stress. However, Nrf2 expression gradually returned to a normal level in the BPA + R30 groups. Because RSV enhances the antioxidant capacity of the liver by inducing Nrf2/HO-1 expression, it is evident that certain properties of RSV were preserved after esterification to RBE, which offset the damaging effects of BPA. Consequently, HO-1 expression could still be observed in the liver tissues of the male offspring rats. This may be attributed to the promotion of HO-1 transcription by Nrf2 after its entry into the cell nucleus, which led to increased protein expression in the liver. In short, the mRNA levels of antioxidant genes, activities of antioxidant enzymes, and observations of angiogenesis and IHC staining of the Nrf2/HO-1 distribution in the liver tissue sections indicate that RBE enhanced the expression of all the selected genes and effectively attenuated extramedullary hematopoiesis and mononuclear cell infiltration.

### 3.3. RBE Exerted Protective Effects against Liver Damage in Male Offspring Rats via Alterations of Gut Microbiota

The gut microbiome is a complex ecosystem, and gut microbiota are closely associated with health and disease. In this study, we studied the effects of RBE, which was denatured into RSV and butyric acid by the gastrointestinal system, to determine whether BPA exposure in maternal rats affected the gut microbiota of the offspring and whether RBE exerted any effects against BPA exposure. The gut microbiota of a healthy individual maintains the functions of the intestinal mucosa and strengthens the human immune system through secretions. Therefore, abnormalities in gut microbiota can damage the intestinal barrier and increase the tendency for bacteria to enter the bloodstream or organs. The liver is particularly susceptible to damage because bacterial translocation mainly occurs through the hepatic portal vein, thereby triggering immune responses and causing oxidative damage [66]. Shukla et al. (2021) proposed the gut–liver–brain (GLB) axis hypothesis and reported that chronic stress and corticosterone can exacerbate alcohol-induced mucosal barrier dysfunction, endotoxemia, and the systemic alcohol response. The authors also demonstrated that gut microbiota modulation probably plays a key role in oxidative stress induced by alcohol [67]. In addition, the metabolites of the gut microbiota, including lipopolysaccharides, ethanol, ammonia, and acetaldehyde, also promote the development of chronic hepatitis [68]. Therefore, researchers have studied the use of probiotics or prebiotics for the regulation of gut microbiota to alleviate the symptoms of non-alcoholic fatty liver disease and other liver diseases [69,70]. These studies have led to a growing interest in the relationship between the gut and the liver, i.e., the gut–liver axis.

In the present study, the abundance of Blautia, Prevotella, Allobaculum, and Lactobacillaceae increased after BPA exposure, with the increase in *B. pseudolongum* and *C. ruminantium* being particularly prominent, whereas the abundance of Adlercreutzia and Oscillospira decreased. After treatment with RBE (BPA + R30), the abundance of *S24-7* and Adlercreutzia increased significantly, whereas the abundance of Allobaculum, Blautia, Lactobacillaceae, and Prevotella decreased significantly; a decrease was also observed in the abundance of *C. ruminantium*. The RBE group demonstrated an increase in *S24-7* abundance and decrease in Lactobacillaceae abundance. Previous studies have indicated that Prevotella may exert pro-inflammatory effects, with increases in the abundance of Prevotella observed in patients with hypertension and new-onset rheumatoid arthritis [71]. Our results also show that *S24-7* was positively correlated with the SCFA concentration in the gut. In addition to serving as a key energy source for colonic epithelial cells, SCFAs also exert protective effects on the intestinal barrier’s function and effectively inhibit inflammatory responses [72]. In addition, Prevotella and *S24-7* demonstrate high glycolytic activities and are mutually exclusive. As shown by previous studies, the direct ingestion of BPA disrupts the gut microbiome. However, this study indicated that the perinatal ingestion of BPA by the maternal rats also disrupted the gut microbiota in the offspring. This may be attributed to the transfer of BPA from maternal rats to offspring through umbilical cord blood during pregnancy or through breast milk during breastfeeding, which enabled BPA to exert its effects on the gut microbiota of the next generation. Additionally, the BPA-induced liver damage suffered by the offspring may also have influenced the gut microbiota distribution via the gut–liver axis.

SCFAs are important microbial metabolites in the gut. In addition to serving as a key source of energy in colonic epithelial cells, they participate in mucoprotein synthesis by activating the AMPK pathway, regulate the oxidation response through the inhibition of histone deacetylases (HDACs), and reduce DNA damage in epithelial cells. Furthermore, butyric acid can also inhibit inflammatory responses by regulating monocyte differentiation via HDAC inhibition and reducing macrophage activity [41]. An increase in the SCFA concentration in the intestine also effectively reduces the risk of constipation and colorectal cancer [73]. In the present study, maternal exposure to BPA increased the acetic acid and propionic acid concentrations in the guts of the offspring. However, no significant difference was observed in the butyric acid content compared with that in the control group. Similarly, previous studies have reported increases in the concentrations of acetic acid and propionic acid in the guts of obese individuals, whereas the concentrations of butyric acid remained constant [74]. BPA + R30 treatment effectively increased the concentrations of acetic acid, propionic acid, and butyric acid in the gut. This may be related to the increase in the abundance of *S24-7* and Adlercreutzia. *S24-7* demonstrates high glycolytic activity and is advantageous for the production of SCFAs [75]. In addition to providing energy to the intestinal epithelial cells, these metabolites can also enter the liver via the portal vein circulation, thereby providing energy and inhibiting inflammation in the liver.

## 4. Materials and Methods

### 4.1. Synthesis of Resveratrol Butyrate Ester

RBE was synthesized according to the method described by Tain et al. [39]. RSV (TCI Development Co., Ltd., Shanghai, China) was mixed with butyric acid (ACROS, New Jersey, USA) in tetrahydrofuran (THF) (Echo Chemical Co., Ltd., Miaoli County, Taiwan). Subsequently, *N*-ethyl-*N*′-(3-dimethylaminopropyl) carbodiimide (EDC) (Sigma-Aldrich, Missouri, USA) and 4-dimethylaminopyridine (DMAP) (Sigma-Aldrich, Missouri, USA) were added, and the esterification reaction was carried out for 48 h in the absence of light. Upon completion of the reaction, a copious amount of distilled water was added, and the reaction mixture was filtered to obtain the precipitated RBE, which was freeze-dried and stored at −20 °C. The novel RBE was produced by the esterification of RSV and butyric acid, including RSV (about 17.11%), RBE monoester (47.12%), and RBE diester (35.00%) [40].

### 4.2. Experimental Animals

**Figure A1:**
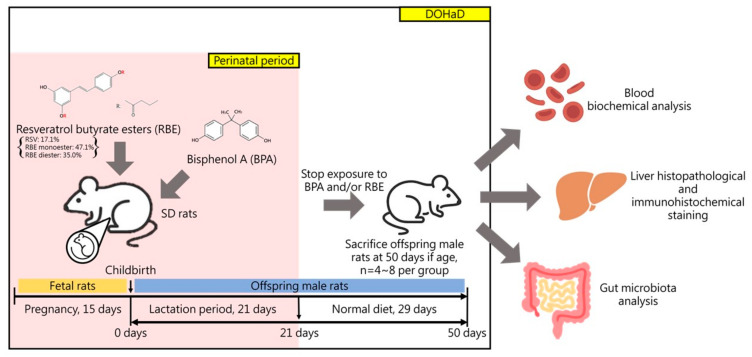
The graphical abstract of this manuscript.

**Figure A2:**
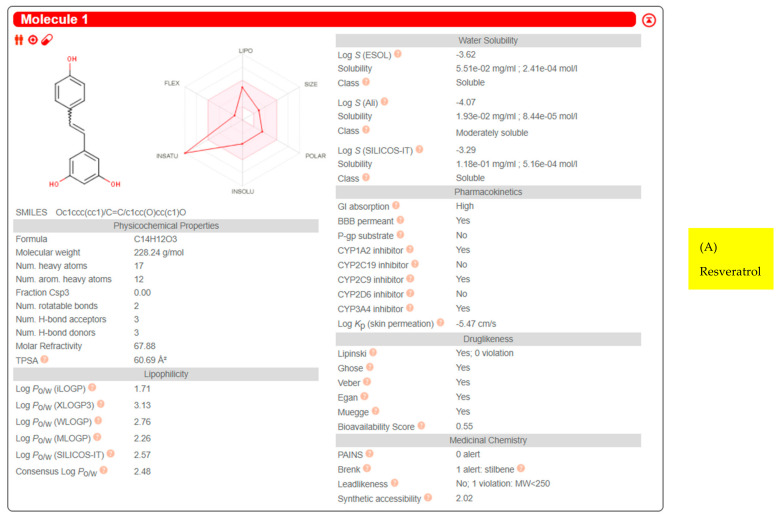

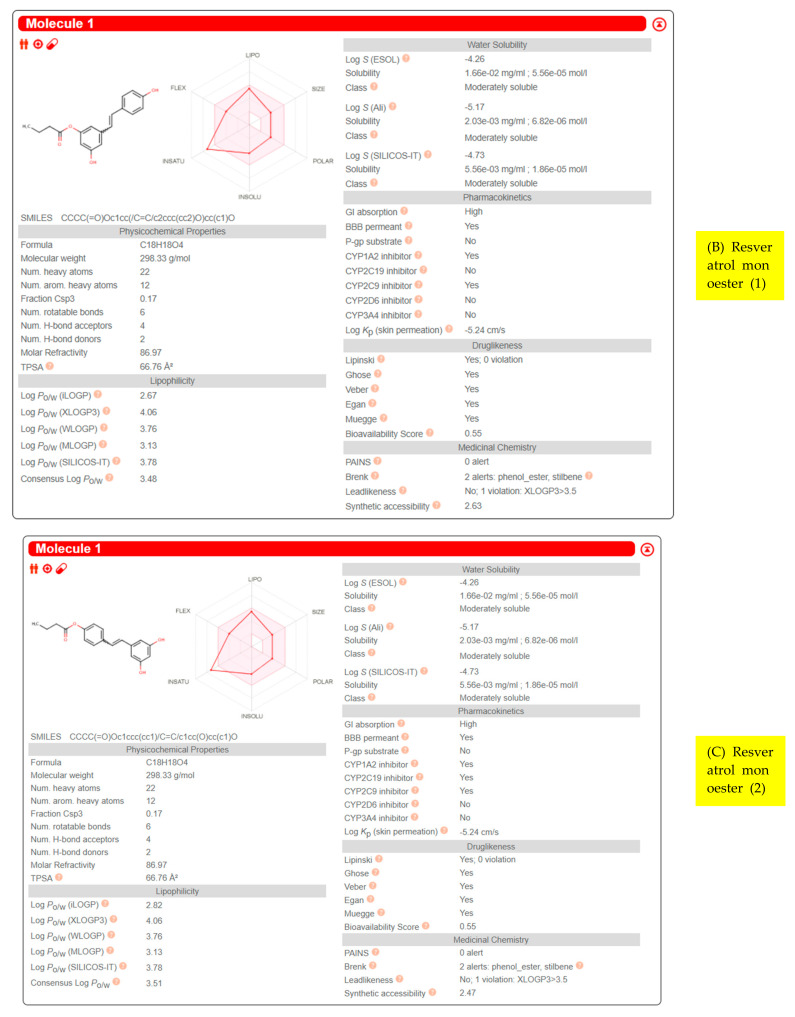

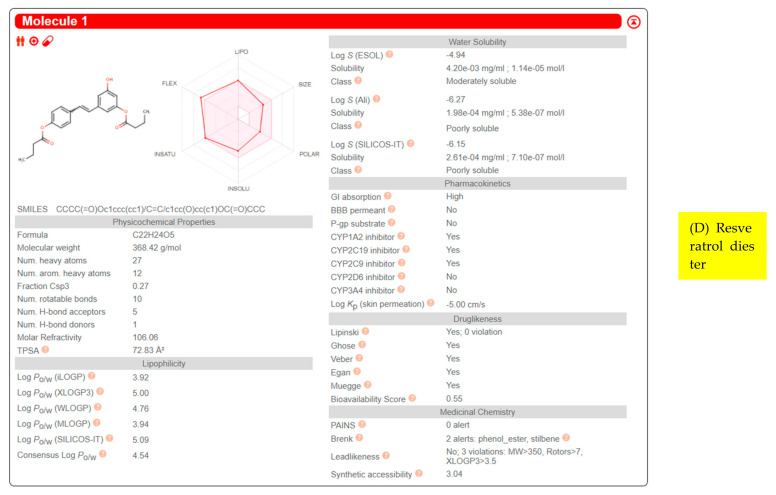
The SwissADME evaluation of (**A**) resveratrol, (**B**) resveratrol mono-ester (1), (**C**) resveratrol mono-ester (2), and (**D**) resveratrol diester (data source: the free SwissADME software http://www.swissadme.ch/index.php, accessed on 20 April 2021).

This experiment was designed by referring to a series of DOHaD trials and proceeded using the 3Rs principles for animal experimentation proposed by Tain et al. [9,10,12,76]. The experimental design is shown in Figure A1. Fifteen-week-old pregnant Sprague Dawley (SD) rats purchased from BioLASCO Taiwan Co., Ltd. (Taipei, Taiwan) were used as experimental models in this study in accordance with the Guide for the Care and Use of Laboratory Animals published by the National Institutes of Health (NIH) in the United States. The study was approved by the Committee for Laboratory Animals of National Kaohsiung University of Science and Technology (Approval No. 0108-AAAP-010, approved 27 Dec 2021). All the animals were reared in an animal facility accredited by the Association for Assessment and Accreditation of Laboratory Animal Care International (AAALAC) with a 12 h light/12 h dark cycle, with the temperature controlled at 25 ± 1 °C. The fifteen-week SD pregnant female rats were assigned to four groups: control (CN); R30, administered 30 mg/kg/day of RBE (dissolved in corn oil); BPA, administered 50 μg/kg/day of BPA (dissolved in corn oil); and BAP + R30, administered 50 μg and 30 mg/kg/day of BPA and RBE, respectively. The dose of BPA used here was based on a previous study [26,77]. RBE is a novel synthesis compound; the dose designed in this study referenced the dose used in RSV related studies [26,28,78]. The pregnant female rats were orally administered BPA and/or RBE from the sixth day of pregnancy for 36 days continuously, including the 21-day lactation period. Subsequently, the male offspring rats (*n* = 4–8 per group) continued to be administered a normal diet (Fwusow Taiwan Co., Ltd., Taichung, Taiwan; 52% carbohydrates, 23.5% protein, 4.5% fat, 10% ash, and 8% fiber) [26] until they were euthanized 50 days after birth. The heparinized blood, liver, and intestinal feces were collected and stored at −80 °C for subsequent analysis. Only male offspring rats were selected from each litter and used in subsequent experiments to avoid experimental errors caused by gender differences.

### 4.3. Plasma Biochemistry Assay

The blood samples of the euthanized offspring rats were centrifuged at 1500× *g* for 10 min at 4 °C, and the supernatant was collected to obtain plasma samples for further experimentation. The TG, TC, HDL, LDL, AST, ALT, and MDA levels in the plasma were measured using commercial assay kits in accordance with the manufacturer’s instructions.

#### 4.3.1. Plasma TG Concentration

Measurements were performed using the Free Fatty Acid Assay Kit (Abcam, Cambridge, UK). The plasma was first diluted with the Fatty Acid Assay Buffer. A total of 2 μL of Acyl-CoA Synthetase reagent was then added to 50 μL of the diluted plasma, and the mixture was maintained at a constant temperature of 37 °C for 30 min. Subsequently, 50 μL of the Fatty Acid Reaction Mix (44 μL of Assay Buffer, 2 μL of Fatty Acid Probe, 2 μL of Enzyme Mix, and 2 μL of Enhancer) was added, and the mixture was maintained at a constant temperature of 37 °C in the absence of light for 30 min. The absorbance was measured at 570 nm and converted to TG concentration using the palmitic acid standard.

#### 4.3.2. Plasma TC Concentration

Measurements were performed using the Cholesterol Assay Kit (Abcam, UK). Plasma was first diluted with the Cholesterol Assay Buffer. A total of 50 μL of the Total Cholesterol Reaction Mix (44 μL of Cholesterol Assay Buffer, 2 μL of Cholesterol Probe, 2 μL of Enzyme Mix, and 2 μL of Cholesterol Esterase) was added to 50 μL of the diluted plasma, and the mixture was maintained at a constant temperature of 37 °C for 60 min. The absorbance was measured at 570 nm and converted to TC concentration using the cholesterol standard.

#### 4.3.3. Plasma HDL and LDL Concentrations

Measurements were performed using the Cholesterol Assay Kit (Abcam, Cambridge, UK). Plasma was first diluted with a double volume of 2X LDL/VLDL Precipitation Buffer. Subsequently, the diluted sample was set aside at room temperature for 10 min and centrifuged at 2000× *g* for 10 min. The HDL-containing supernatant was transferred to a new centrifuge tube, and the LDL-containing sediment was diluted with 200 μL of PBS. A total of 50 μL of the Total Cholesterol Reaction Mix (44 μL of Cholesterol Assay Buffer, 2 μL of Cholesterol Probe, 2 μL of Enzyme Mix, and 2 μL of Cholesterol Esterose) was added to 50 μL of the supernatant and diluted sediment, and the mixtures were maintained at a constant temperature of 37 °C for 60 min. The absorbance was measured at 570 nm, and the HDL concentration in the supernatant and LDL concentration in the sediment were obtained by conversion using the Cholesterol Standard.

#### 4.3.4. Plasma AST Activity

Measurements were performed using the AST/GOT Reagent (Thermo Fisher Scientific, Waltham, MA, USA). A total of 30 μL of plasma was first added to 300 μL of AST/GOT Reagent and mixed for 30 s. The absorbance was measured after 1, 2, and 3 min at 340 nm, and the AST activity was calculated using the following formula:

AST activity = Asample/min × (0.33 × 1000) / (6.3 × 0.03 × 10)

#### 4.3.5. Plasma ALT Activity

Measurements were performed using the ALT/GPT Reagent (Thermo Fisher Scientific, Waltham, MA, USA). A total of 30 μL of plasma was first added to 300 μL of ALT/GPT Reagent and mixed for 30 s. The absorbance was measured after 1, 2, and 3 min at 340 nm, and ALT activity was calculated using the following formula:

ALT activity = Asample/min × (0.33 × 1000) / (6.3 × 0.03 × 10)

#### 4.3.6. Plasma MDA Concentration

Measurements were performed using the Lipid Peroxidation (MDA) Assay Kit (Abcam, Cambridge, UK). A total of 20 μL of plasma was added to 500 μL of 42 mM H_2_SO_4_, followed by the addition of 125 μL of the phosphotungstic acid solution. The mixture was set aside for 5 min before centrifugation at 12,000× *g* for 3 min. The sediment was collected and diluted in 200 μL of distilled water, followed by the addition of 600 μL of TBA Reagent. The mixture was maintained at 95 °C for 60 min. After cooling in an ice bath for 10 min, 200 μL of the supernatant was collected for measuring the absorbance at 532 nm, and the MDA concentration was determined by conversion using the MDA standard.

### 4.4. Gene Expression Analyses

RNA extraction was performed using the Total RNA Miniprep Purification Kit (GMbiolab, Taichung, Taiwan). RNA was reverse-transcribed into cDNA using M-MLV Reverse Transcriptase with oligo dT as a primer. Subsequently, five antioxidant target genes were analyzed: *Sod1*, *Sod2*, *Gpx1*, *Gpx2*, and *catalase*. The GAPDH gene was used as an internal control gene. The primer sequences are listed in Table 3. The RNA expression levels were normalized to the GAPDH mRNA levels and calculated according to the ΔCt method.

### 4.5. Analyses of Antioxidant Enzyme Activity in the Liver

#### 4.5.1. SOD Activity Analyses

The SOD activity in the liver tissue was measured using the Superoxide Dismutase Assay Kit (Cayman Chemical, Ann Arbor, MI, USA). A total of 0.1 g of liver tissue was homogenized with 1 mL of HEPES buffer (20 mM HEPES, 1 mM EGTA, 210 mM Mannitol, 70 mM Sucrose, pH 7.2), and the mixture was centrifuged at 12,000× *g* for 15 min at 4 °C. The supernatant was collected and diluted with the sample buffer. A total of 10 μL of the diluted supernatant was obtained for the addition of 200 μL of the Radical Detector followed by 20 μL of xanthine oxidase, and the mixture was set aside for 30 min. The absorbance was measured at 450 nm and converted to SOD activity using the SOD standard.

#### 4.5.2. GSH Concentration Analyses

A total of 0.1 g of liver tissue was homogenized with 0.3 mL of 2% TCA, stored at 4 °C for 10 min, and centrifuged at 12,000× *g* for 15 min. The supernatant was collected, diluted tenfold with distilled water, and subjected to HPLC analysis by cold injection using the following parameters: mobile phase—5% acetonitrile, 0.1% trifluoroacetic acid, 1.2% sodium perchlorate; flow rate—1 mL/min; column—Zorbax 5 μm C18, 250 × 4.6 mm; and detection wavelength—220 nm.

#### 4.5.3. CAT Activity Analyses

The liver tissue specimen was homogenized with a tenfold volume of 0.05 M PBS at pH 7.0. Subsequently, 50 μL of the supernatant was added to 100 μL of H_2_O_2_ reagent (10 mM H_2_O_2_ in PBS) and reacted at 37 °C for 20 min. Subsequently, 100 μL of AMR reagent (0.01 M Ammonium in 0.5 M H_2_SO_4_) was added, and the mixture was set aside for 10 min. The absorbance was measured at 452 nm, and the CAT activity was analyzed using the CAT standard.

### 4.6. Histopathology Staining

The liver tissue specimen obtained from each rat was fixed in 10% formalin, washed and dehydrated through graded ethanol series, rinsed in xylene, embedded in paraffin wax, and sectioned into approximately 5 μm thick slices. Subsequently, the slices were washed thrice with xylene (10 min each) and twice with anhydrous alcohol (5 min each), rehydrated via graded alcohol series (95% alcohol for 2 min, 85% alcohol for 2 min, and 75% alcohol for 2 min), rinsed briefly with water, and stained with Mayer’s hematoxylin solution for 1 min. The stained slices were rinsed with tap water for 3 min, counterstained with eosin solution for 5 min, dehydrated with 95% alcohol and twice with anhydrous alcohol (5 min each), and cleared twice in xylene (5 min each). Images of the stained slices were captured using a MoticEasyScan system and analyzed using the Motic DS Assistant (4K).

### 4.7. Immunocytochemistry Staining

The liver tissue specimen of each rat was fixed in 10% formalin, washed and dehydrated through graded alcohol series, rinsed in xylene, embedded in paraffin wax, and sectioned into approximately 5 μm thick slices. Subsequently, the slices were washed thrice with xylene (10 min each time) and twice with anhydrous alcohol (5 min each), rehydrated via graded alcohol series (95% alcohol for 2 min, 85% alcohol for 2 min, and 75% alcohol for 2 min), and washed twice with PBS. Immunocytochemical staining was performed using the UltraVision™ Quanto Detection System HRP DAB (Thermo Fisher Scientific, Waltham, MA, USA). First, the slices were incubated in hydrogen peroxide block for 10 min, and heat-induced antigen retrieval was performed for 15 min using the citrate buffer solution (pH 6.0). Subsequently, the slices were incubated in Ultra V Block for 5 min. The slices were then incubated with the primary antibody (Nrf2 or HO-1) at 37 °C for 1 h, washed twice with PBS, and incubated with the secondary antibody at 37 °C for 1 h. After adding 30 µL of DAB Quanto Chromogen to 1 mL of DAB Quanto Substrate, the slices were soaked in the resultant solution for 10 min and subsequently stained with Mayer’s hematoxylin solution for 1 min. The stained slices were rinsed with tap water for 3 min, counterstained with eosin solution for 5 min, dehydrated with 95% alcohol and twice with anhydrous alcohol (5 min each time), and cleared twice in xylene (5 min each time). The antibodies used for immunostaining were as follows: Anti-Heme Oxygenase 1 antibody (ab13243) (Abcam, Cambridge, UK), Rabbit NRF2 antibody [N2C2] (GenTex, Zeeland, MI, USA), and Goat anti-Rabbit IgG (H + L) Secondary Antibody, HRP (Thermo, Waltham, MA, USA). Images of the stained slices were captured using a MoticEasyScan system and analyzed using the Motic DS Assistant (4K).

### 4.8. Next-Generation Sequencing (NGS) Analysis

Genomic DNA was extracted from the fecal samples using the QIAmp Fast DNA Stool Mini Kit (Qiagen, Hilden, Germany) according to the standard instructions. The concentration was assessed using a NanoDrop 2000 (O.D 260/280: 1.7–2.2; conc. > 50 ng/µL), and the DNA was diluted tenfold (final conc.: 4–6 ng/µL) with elution buffer.

The library was constructed with the standard V3–V4 region of the 16S rRNA gene. PCR amplification was performed with the KAPA HiFi Hotstart® readymix (Roche, Basel, Switzerland), and the products were purified with AMPure XP magnetic beads (Beckman Coulter, Brea, CA, USA). The amplification and quality of the PCR product were assessed using a Fragment Analyzer (Advanced Analytical, Parkersburg, WV, USA), and the product was quantified using a Qubit 3.0 Fluorometer. Then, the library was sequenced using a MiSeq sequencer (Illumina, San Diego, CA, USA) with paired-end reads (2 × 300 nt) and at least 100,000 reads per sample.

### 4.9. Bioinformatics Analysis and Statistics

The raw paired-end reads were trimmed, and those passing the quality filters were assigned to operational taxonomic units (OTUs) that had ≥97% similarity to entries in the GreenGene Database (v. 13.8). The OTU taxonomy (relative abundance, heatmap), alpha diversity (Shannon index, Venn diagram), and beta diversity (PCoA, phylogenetic curve) were determined using the Basespace (Illumina, San Diego, CA, USA), CLC genomics workbench (Qiagen, Hilden, Germany), and GraphPad Prism 8 (GraphPad Software, USA). The core bacteria analysis (linear discriminant analysis effect size, LEfSe) and functional analysis (Phylogenetic Investigation of Communities by Reconstruction of Unobserved States, PICRUSt) were performed using the Galaxy/Hutlab website (http://huttenhower.sph.harvard.edu/galaxy/, accessed on 31 January 2021). A *p*-value less than 0.05 was considered statistically significant.

### 4.10. SCFA Measurement in the Feces and Analysis by GC–MS

A total of 0.03 g of rat feces was weighed and homogenized in 0.3 mL of deionized water. After centrifugation, 150 μL of the supernatant was obtained and acidified using 50 μL of sulfuric acid. After 10 μL of 2-ethylbutyric acid was added to serve as the internal standard, 400 μL of diethyl ether was added, and the mixture was shaken for 15 min. Subsequently, the mixture was centrifuged at 9000× *g* for 10 min at 4 °C, and the supernatant was obtained for GC/MS analysis. The chromatography column was a DB-FFAP capillary column (30 m × 0.25 mm × 0.25 µm). The flow rate of the mobile phase gas (helium) was 1 mL/min. The heating was maintained as follows. The initial temperature of 80 °C was maintained for 1 min before increasing to 240 °C at a rate of 10 °C/min, which was maintained for 12 min. The injector and transfer line temperatures were set at 240 °C, the ion source (70 eV) temperature was 230 °C, the quadrupole temperature was 150 °C, and the scanning range was 35–550 m/z. The mass spectrum of the identified compound was compared with the GC/MS database to confirm the compound type. Each peak identified by MS spectrum matching had a corresponding retention time (RT) and qualitative analysis (QUAL) comparison rate.

### 4.11. Statistical Analyses

All the experiments were performed at least twice, and three samples were used for each test. Data were collected and analyzed using one-way analysis of variance (ANOVA) and Duncan’s test. Significant differences were noted when *p* < 0.05. All the statistical analyses were performed using SPSS (version 12.0, St. Armonk, NY, USA). The ADME of resveratrol and RBE were evaluated using the SwissADME free web tool (http://www.swissadme.ch/index.php, accessed on 20 April 2021), which can be used to evaluate pharmacokinetics, drug-likeness, and medicinal chemistry friendliness.

## 5. Conclusions

In the present study, we found that RBE effectively attenuated BPA-induced oxidative damage in the liver, reduced ALT and AST activities, and effectively increased the mRNA expression and activities of antioxidant enzymes, thereby enhancing liver function and reducing oxidative damage in the livers of offspring rats. The results of tissue staining revealed clearly visible liver damage induced by BPA, which was alleviated after treatment with RBE. Our results also show that the ingestion of BPA by maternal rats resulted in abnormalities in the gut microbiota distribution of their offspring, which decreased the intestinal barrier function and resulted in the development of inflammatory responses and oxidative damage in the livers of the offspring. However, the administration of RBE effectively increased the abundance of *S24-7* and Adlercreutzia in the liver and the concentration of butyric acid in the gut, which demonstrated reparative effects on the colonic epithelial cells, in addition to neuroprotective effects. These results demonstrate the potential applications of RBE in the development of liver-protecting drugs that exert preventive effects against BPA-induced oxidative damage.

## Figures and Tables

**Table 1:** Plasma biochemical parameters of male offspring rats.

Groups *	CN	R30	BPA	BPA + R30
Body weight (g)	246.6 ± 8.1 ^a^	293.2 ± 1.3 ^b^	276.5 ± 5.2 ^c^	240.1 ± 8.6 ^a^
ALT (U/L)	49.5 ± 2.9 ^a^	58.4 ± 3.4 ^b^	86.5 ± 6.5 ^c^	51.5 ± 6.8 ^ab^
AST (U/L)	102.5 ± 9.7 ^a^	140.8 ± 23.5 ^b^	204.0 ± 21.3 ^c^	101.7 ± 10.5 ^a^
TG (μg/mL)	60.0 ± 13.1 ^a^	101.0 ± 17.2 ^b^	160.0 ± 8.6 ^c^	93.2 ± 13.4 ^b^
TC (μg/mL)	1283.6 ± 12.5 ^a^	1270.1 ± 21.9 ^a^	1357.6 ± 19.5 ^b^	1244.2 ± 4.2 ^a^
HDL (μg/mL)	193.3 ± 8.3 ^a^	197.1 ± 2.0 ^a^	71.7 ± 16.9 ^b^	238.9 ± 4.6 ^c^
LDL (μg/mL)	592.5 ± 78.2 ^a^	586.0 ± 57.9 ^a^	726.3 ± 55.8 ^b^	575.3 ± 57.4 ^a^
MDA (pg/mg)	248.5 ± 7.7 ^a^	117.6 ± 0.9 ^b^	308.4 ± 20.4 ^c^	173.1 ± 7.7 ^d^

* *n* = 4–8 per group; ^a–d^ *p* < 0.05, significantly different from other groups. CN = control group; R30 = group administered 30 mg/kg/day of resveratrol butyrate ester (RBE); BPA = group with bisphenol A at 50 μg/kg/day; BPA + R30 = group administered 30 mg/kg/day of RBE and bisphenol A.

**Table 2:** Short-chain fatty acid (SCFA) concentrations in feces of male offspring rats.

Feces, SCFAs (μmol/g Feces)	CN	R30	BPA	BPA + R30
Acetic acid	13.52 ± 1.78 ^a^	23.09 ± 1.80 ^b^	28.56 ± 5.58 ^b^	25.49 ± 1.73 ^b^
Propanoic acid	3.56 ± 0.46 ^a^	5.06 ± 0.56 ^ab^	5.68 ± 0.59 ^b^	5.86 ± 0.77 ^b^
Butanoic acid	3.96 ± 0.49 ^a^	3.86 ± 0.42 ^a^	5.01 ± 0.55 ^a^	7.13 ± 0.44 ^b^

^a–b^ *p* < 0.05, significantly different from other groups. *n* = 4–8 per group; CN = control group; R30 = group administered 30 mg/kg/day of RBE; BPA = group administered bisphenol A at 50 μg/kg/day; BPA + R30 = group administered 30 mg/kg/day of RBE and bisphenol A.

**Table 3:** mRNA primer sequences.

Gene	Forward	Reverse
*Sod1*	CCACTGCAGGACCTCATTTT	CACCTTTGCCCAAGTCATCT
*Sod2*	CCGAGGAGAAGTACCACGAG	GCTTGATAGCCTCCAGCAAC
*Gpx1*	TGAGAAGTGCGAGGTGAATG	AACACCGTCTGGACCTACCA
*Gpx2*	GACACGAGGAAACCGAAGCA	GGCCCTTCACAACGTCT
*Catalase*	ACATGGTCTGGGACTTCTGG	CAAGTTTTTGATGCCCTGGT
*GAPDH*	ATGGGAAGCTGGTCATCAAC	GTGGTTCACACCCATCACAA

## Data Availability

Not applicable.

## Appendix A

**Table A1:** The SwissADME evaluation of resveratrol, resveratrol mono-ester (1), resveratrol mono-ester (2), and resveratrol diester.

Compounds	Structure	Pharmacokinetics *				
		GI Absorption	BBB Permeant	P-gp Substrate	Log Kp (Skin Permeation)	CYP-Family Inhibitor
Resveratrol		High	Yes	No	−5.47 cm/s	Some yes, some no
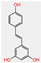
Resveratrol mono-ester (1)		High	Yes	No	−5.24 cm/s	Some yes, some no
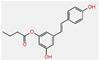
Resveratrol mono-ester (2)		High	Yes	No	−5.24 cm/s	Some yes, some no
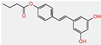
Resveratrol diester		High	No	No	−5.00 cm/s	Some yes, some no
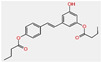

* Data source: the free SwissADME software http://www.swissadme.ch/index.php, accessed on 20 April 2021.
